# Temporal and Spatial Variation of Soil Bacteria Richness, Composition, and Function in a Neotropical Rainforest

**DOI:** 10.1371/journal.pone.0159131

**Published:** 2016-07-08

**Authors:** Stephanie N Kivlin, Christine V Hawkes

**Affiliations:** Department of Integrative Biology, University of Texas at Austin, Austin, TX 78712, United States of America; National Taiwan University, TAIWAN

## Abstract

The high diversity of tree species has traditionally been considered an important controller of belowground processes in tropical rainforests. However, soil water availability and resources are also primary regulators of soil bacteria in many ecosystems. Separating the effects of these biotic and abiotic factors in the tropics is challenging because of their high spatial and temporal heterogeneity. To determine the drivers of tropical soil bacteria, we examined tree species effects using experimental tree monocultures and secondary forests at La Selva Biological Station in Costa Rica. A randomized block design captured spatial variation and we sampled at four dates across two years to assess temporal variation. We measured bacteria richness, phylogenetic diversity, community composition, biomass, and functional potential. All bacteria parameters varied significantly across dates. In addition, bacteria richness and phylogenetic diversity were affected by the interaction of vegetation type and date, whereas bacteria community composition was affected by the interaction of vegetation type and block. Shifts in bacteria community richness and composition were unrelated to shifts in enzyme function, suggesting physiological overlap among taxa. Based on the observed temporal and spatial heterogeneity, our understanding of tropical soil bacteria will benefit from additional work to determine the optimal temporal and spatial scales for sampling. Understanding spatial and temporal variation will facilitate prediction of how tropical soil microbes will respond to future environmental change.

## Introduction

Soil microbial communities in tropical ecosystems typically are assumed to track plant species and plant diversity [1–5]. Indeed, there is evidence that tropical soil microbial biomass positively tracks plant richness [3] and community composition of both bacteria [4] and fungi [6] shift when tropical plant diversity is reduced by land use change. Limited soil resources in tropical soils [7] may mean that soil microorganisms rely more on plants for carbon and nutrients via rhizosphere exudation or litter production, which varies among plant species [8–10]. But, plant effects are not always predominant. In the most comprehensive observational survey to date, bacteria and fungal community composition were only slightly correlated with plant community composition, and varied more with soil pH [1].

Function in soil microorganisms can respond to the same factors as community composition, but this is not necessarily the case. Extracellular enzymes are responsive to resource quality and quantity [11], soil pH [12], and soil heavy metal content [13], all of which can vary in soils conditioned by different tree species. However, enzymes are also responsive to factors such as soil moisture and temperature [14, 15] or texture [16], which may vary more temporally or spatially instead of with tree species. Moreover, the link between composition and function is not always straightforward because microbial taxa exhibit a high degree of functional redundancy [17].

The uncertainty of plant effects on microbial community composition and function may stem from the immense spatial and temporal heterogeneity in tropical plants and their inputs [18]. For example, variation across tropical landscapes in soil nitrogen, phosphorus, and cations has been observed from two- to 19-fold [19]. Moreover, soil resources, cycling rates, and enzymes in three tropical forests were primarily explained (>61%) by variation at scales of 1-m^2^ [20]. Soil phosphorus concentrations are also temporally variable over short time periods, correlated with antecedent rainfall [21]. Moisture can restrict microbial activity both directly [22] and via access to dissolved substrates [23], but in some cases moisture is decoupled from both microbial functions and biogeochemical cycles [24]. Separation of tree species effects from spatial and temporal heterogeneity in resources and moisture is thus needed to parse their relative importance.

We addressed how soil bacteria were affected by tree species and abiotic factors in a wet Neotropical rainforest by sampling soils in experimental monoculture plots of four native trees (*Hyeronima alchorneoides*, *Pentaclethra macroloba*, *Virola koschnyi*, *Vochysia guatemalensis*) at La Selva Biological Station in Costa Rica [25]. We also included secondary forest plots as controls, which were established and naturally regenerated at the same time as the experimental plots. The randomized block design allowed us to examine spatial variation in soil properties. To capture temporal variation, we sampled the plots in wetter and drier seasons across two years. In each sample, we measured soil bacteria abundance, richness, phylogenetic diversity, community composition, and function (enzyme activities). This approach allowed us to separate temporal and spatial effects from those caused by tree species identity, which is not possible in gradient or mixed-forest studies.

We hypothesized that bacterial richness, phylogenetic diversity, community composition, and function would all respond similarly to the experimental tree species stands based on prior studies that demonstrated differences among trees in soil carbon storage, plant inputs, nitrogen-fixation, and fungal composition [26–28]. Among the tree species, we expected that stands of the nitrogen-fixing *Pentaclethra* would differ from all others based on the potential for both resource effects and biotic interactions with *Rhizobium* bacteria. We further expected that, if bacteria respond to belowground carbon inputs, community composition and function would vary between *Hyeronima* and *Virola* stands, with *Vochysia* stands intermediate based on observed variation in soil carbon inputs among these species [28]. Finally, if tree effects are additive, we expected secondary forests would be intermediate among other stands because they contain a mixture of all four tree species, as well as others. Block and date effects would indicate spatial and temporal heterogeneity, respectively, and we expected these to correlate with underlying variation in soil nutrients or soil moisture that were measured but not manipulated in the present study.

## Methods

### Study site and experimental design

The experiment was located at La Selva Biological Station, Costa Rica (10°25'53.14''N, 84°0'10.51''W). Mean annual precipitation is 4,142 mm and varies two-fold between wetter months of June-November and drier months of January-April; temperature is consistently 25°C year round (Organization for Tropical Studies Meteorological Database, 1992–2006). Soils are volcanically derived Ultisols [29]. We sampled soil in both wetter (September 2012, 2013) and drier (February 2013, 2014) months over two years, which varied 3-4x in short-term rainfall (1 week and 1 month prior) and 1.3–2.2x for rainfall 3 to 6 months prior to sampling (Organization for Tropical Studies Meteorological Database, annual observations from 1963–2013, n = 51; S1 Table). Soils were collected under Conagebio permit No. R-005-2012.

Soils were sampled from monoculture stands of four tree species: *Hyeronima alchorneoides* Allemao (Phyllanthaceae), *Pentaclethra macroloba* (Willd.) Ktze (Fabaceae), *Virola koschnyi* Warb. (Myristicaceae), and *Vochysia guatemalensis* J.D. Smith (Vochysiaceae). The trees were established in 1988 in four replicate blocks located 50–200 m apart, each containing 50 m x 50 m tree plots separated by 5 m buffer areas. All blocks were placed on former pastureland, which had been grazed for approximately 30 years [25]. We also sampled secondary forest plots that were also established in 1988 as part of the block design by allowing the pasture to naturally regenerate. During the most recent survey in 2004, the secondary forest plots contained 32 woody plant species including each of the tree species in the monoculture stands except *Virola koschnyi* [30]. One plot of *Vochysia* was struck by lightning and the resulting fire destroyed the plot, allowing only 3 blocks to be sampled for that species. A detailed description of this site can be found in [9]. For convenience, we will refer to the plant species monocultures and secondary forests as “vegetation types.” At each sampling date, we collected five soil cores (10 cm deep x 2.5 cm wide) in each plot by randomly positioning one core in each of the four plot quadrants, collecting a fifth core from a random position in the full plot, and homogenizing the cores into a single plot-level sample. Thus, there were 19 soil samples per date (5 vegetation types x 4 blocks, minus 1 plot for *Vochysia*) and 76 samples total over two years. Soil pH and nutrients (PO_4_^3-^, NH_4_^+^, NO_3_^-^) were measured as in [27]. Sampling across dates and blocks was sufficient to generate variation ranging from 0.7x (moisture) to 83x (nitrate) (S2 Table).

### Bacteria abundance

To estimate abundance at each sampling point, we extracted microbial biomass via chloroform fumigation and extraction with 0.5M K_2_SO_4_ [31] and quantification of extracted carbon by combustion (Apollo 9000 Total Organic Carbon Analyzer; Teledyne-Tekmar, Mason, OH, USA). Microbial biomass carbon was determined to primarily reflect bacteria by accounting for the proportion that was fungal based on soil hyphal lengths following [32]. Briefly, fungal hyphal length (L) (data reported in [27]) was transformed to biovolume using the equation for volume (V) of a cylinder: V = π * r^2^ * L. We assumed a hyphal radius (r) of 1.1 μm [33], fresh tissue density of 1.1 g cm^-3^, 40% solid content, and 40% carbon content [34]. On average, fungal biomass carbon accounted for 2.62% of the total microbial biomass pool and never more than 16%. Furthermore, the fungal fraction of microbial biomass alone was never correlated with the activity of any enzyme (data not shown). We also did not account for Archaea or other soil organisms because we used bacteria-specific PCR primers and did not microscopically identify organisms other than fungi.

### Bacteria community composition

We extracted DNA from two, ~0.25 g samples of soil from each plot at each date using the MoBio PowerSoil kit (MoBio, Carlsbad, CA). DNA was quantified using a Qubit Fluorometer, combined at the plot level, and standardized to ~10 ng μl^-1^ for each sample. A ~450b region of 16S bacteria DNA from each sample was then amplified with primers that consisted of Illumina TruSeq V3 indices (Illumina, San Diego, CA) ligated to 454 MID barcodes (Roche, Basel, Switzerland) linked to bacteria-specific S-D-Bact-0341-b-S-17/S-D-Bact-0785-a-A-21 primers [35]. Each 26.5 μl reaction contained: 21.5 μl of Platinum PCR Supermix (Invitrogen, Carlsbad, CA), 1.25 μl of each primer (10 μM), 0.5 μl of BSA (20 mg ml^-1^), and 2 μl (~20 ng) of DNA. The reactions ran with a hot start at 95°C for 5 min, 25 cycles of 95°C for 40 s, 55°C for 2 min, 72°C for 60 s, and a final extension step of 72°C for 7 min. For each sample, PCR reactions were run in triplicate, combined, and cleaned with Agencourt AMPure XP magnetic beads in a 1.8 ratio of beads to PCR product (Beckman Coulter, Brea, CA), and quantitated with a Qubit fluorometer. Samples were then pooled in equal amounts into four libraries (one for each date) and sequenced as 2 x 300 bp reads on one lane of an Illumina MiSeq v3 sequencer at the University of Texas Genome Sequencing and Analysis Facility.

We obtained ~15.5 million total sequences. Forward and reverse sequences were concatenated using the Mothur v.1.33.3 pipeline [36]. Sequences were then quality filtered using default settings in the QIIME pipeline v. 1.8.0–20140103 [37]. Sequences were discarded if they were less than 300 bases in length, had more than six ambiguous bases, or contained any ambiguous bases in their barcode. Chimeras were removed using the UCHIME algorithm with default parameters [38]. This excluded ~60% of the run leaving ~6.5 million sequences. Sequences were clustered into operational taxonomic units (OTUs) at a 97% similarity cutoff using UCLUST with default parameters [39]. We discarded dataset-wide singletons and OTUs that only occurred in one sample, as these are likely to be sequencing artifacts [40]. We then used the Ribosomal Database Project (RDP) classifier against the RDP 16S bacteria database to assign bacteria taxonomy to each OTU. Non-bacteria OTUs (~9% of OTUs) were discarded, leaving 4.2 million full-length bacteria sequences that were assigned to the phylum-level.

Samples were normalized with the DeSeq2 algorithm [41], which is the most sensitive normalization technique available across a range of sample sizes [42]. An alignment and maximum likelihood phylogeny were created with one representative sequence from each of the 106, 539 bacteria OTUs using Practical Alignment using SATé and TrAnsitivity (PASTA) [43] with the MAFFT aligner [44], OPAL merger [45], and FASTTREE tree estimation [46] algorithms. Sequences were deposited in the NCBI Sequence Read Archive (SRA) under accession number (SRX766152).

### Soil enzyme activities

We measured a broad range of potential soil activities for six C, N, and P-acquiring extracellular, hydrolytic enzymes. These enzymes can be produced by both soil bacteria and fungi [47]. In the current study, we assumed that bacteria were the main producers of extracellular enzymes because bacterial biomass was at least 6x and often 50x greater than fungal biomass. Of course, abundance does not necessarily translate directly to activity [48] and we acknowledge that future studies will be needed to better understand the relative contributions of bacteria and fungi to enzyme activity in tropical soils. We targeted C-degrading enzymes for starch byproducts (alpha-glucosidase; AG), cellulose and glucose byproducts (beta-glucosidase; BG), xylose (beta-xylosidase; BX), and cellulose (cellobiohydrolase; CBH), as well as enzymes relevant for N cycling (N-acetyl-glucosaminidase; NAG) and P cycling (acid phosphatase; AP). Our methods follow [49]. Briefly, for each assay, ~2 g of field-wet soil was blended in a sodium acetate buffer (pH = 5) for 2 min, incubated with the appropriate fluorescently-labeled substrate for 1 h, and measured fluorometrically (λ_ex_ = 365 nm, λ_em_ = 450 nm; Flexstation 3, Molecular Devices, Sunnyvale, CA).

### Statistics

Bacteria diversity was calculated as taxonomic richness (alpha diversity) and phylogenetic diversity using Faith’s PD because it is the most widely used metric for phylogenetic alpha diversity and performs similarly to other metrics [50]. Faith’s PD was calculated as the sum of branch lengths connecting all the species in the bacteria communities in each sample at each date using an ultrametric phylogeny. Bacteria biomass, richness, PD, and potential enzyme activities were analyzed for the entire bacteria community and each phylum that occurred in all samples using linear mixed models (LMMs) with REML estimation as a function of vegetation type, date, block, and their interactions. Date was a repeated measure, vegetation type was a fixed factor, and block was a random factor. We also included the following covariates: soil PO_4_^3-^, NH_4_^+^, NO_3_^-^, pH, soil moisture, and 1-week, 1-month, 3-month, and 6-month antecedent rainfall. Potential covariates were included in the model in decreasing order of their individual Pearson correlation values (S1 Table). When two variables were correlated with each other by over 75%, only the covariate with the most significant correlation to the dependent variable was included. Nevertheless, none of these continuous variables explained any residual variation. When categorical variables were significant at *P* < 0.05 in the LMM, we calculated their partial R^2^ following [51]. When date was a significant effect, we analyzed differences among dates with one-way ANOVAs and Sidak post-hoc tests.

Variation in bacteria community composition was examined as a function of vegetation type, sampling date, block, vegetation type interactions with both date and block, and the same potential covariate pool as above with PERMANOVA [52] using Bray-Curtis dissimilarity of bacteria OTUs. Covariates (soil pH, PO_4_^3-^, and NO_3_^-^) were chosen with the bioenv function in R v. 3.2.1 [53] following [54]. When the main effects were significant, pairwise comparisons of all vegetation types and sampling dates were used to determine significant post-hoc differences among groups. Bacteria community composition was visualized using nonmetric-multidimensional scaling (NMS). We also regressed NMS axis scores against all potential covariates to identify variables that were related to shifts in microbial community composition (S3 Table).

All community statistics were performed in R v. 3.2.1 using the Vegan v. 2.2.0 [55] and Picante v. 1.6–2 [56] packages. LMM and ANOVA statistics were carried out in SPSS v. 17 (IBM, Armonk, New York). Bonferroni corrections [57] were used for multiple comparisons to maintain the family-wise error rate at α = 0.05.

## Results

### Microbial biomass

**Fig 1:**
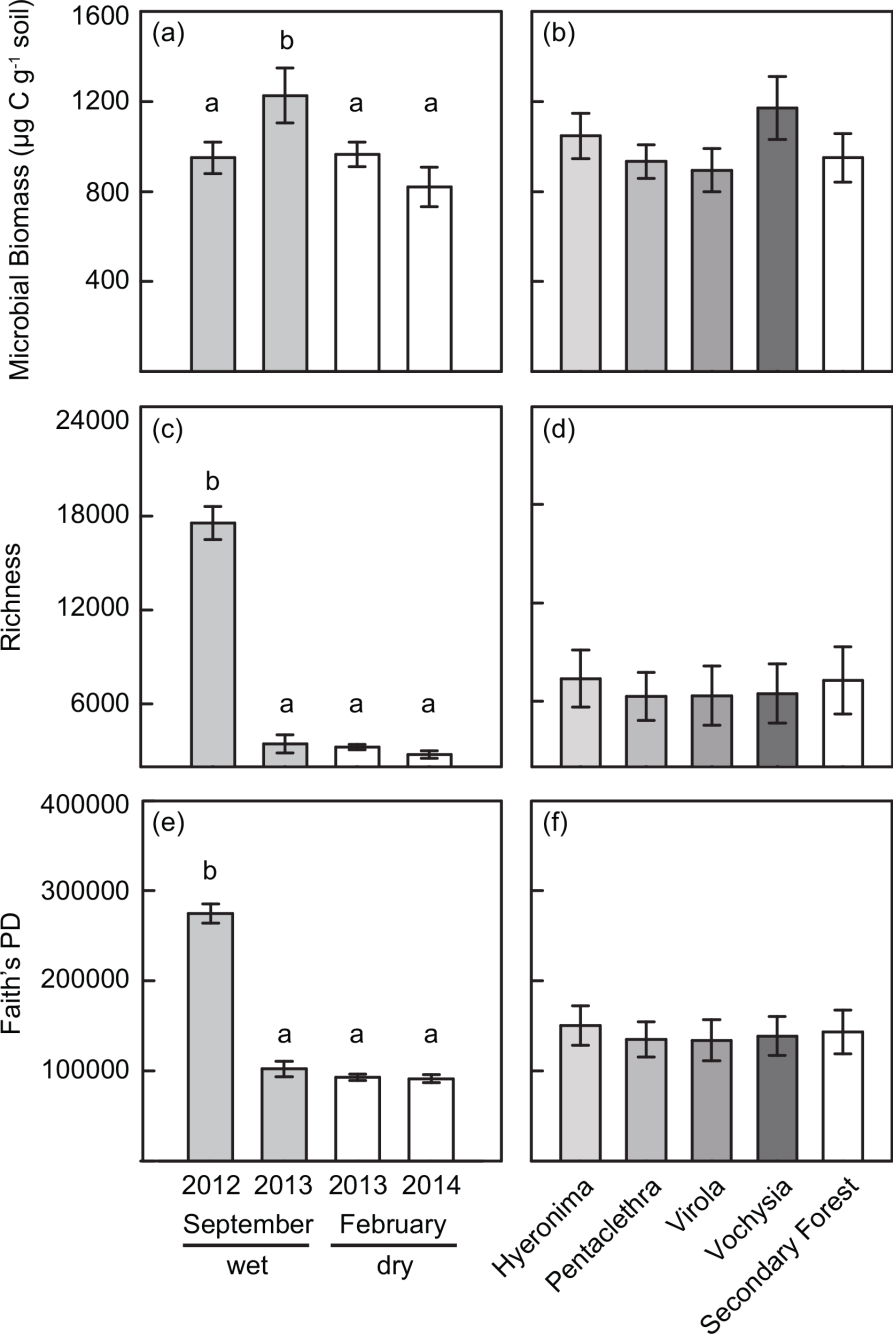
Effects of date and vegetation type on microbial biomass and diversity. (a-b) Microbial biomass, (c-d) bacteria taxonomic richness, and (e-f) Faith’s PD by sampling date and vegetation type. Letters indicate significant differences among treatments in posthoc tests (*P* < 0.05 after Bonferroni correction). Error bars are ± 1 SE (n = 16 for all vegetation types except *Vochysia* where n = 12; for dates n = 19).

Microbial biomass varied among sampling dates (*P* < 0.001; Fig 1A, Table 1), peaking at the wettest of the four dates in September 2013 (S3 Table).

**Table 1:** Linear mixed model analyses of microbial biomass, richness, and PD.

	Biomass			Bacteria richness			Bacteria PD		
	*F* or *Wald’s Z*^a^	*r*^*2*^	*P*^b^	*F* or *Wald’s Z*	*r*^*2*^	*P*	*F* or *Wald’s Z*	*r*^*2*^	*P*
Veg type	0.62	–	0.66	0.21	–	0.99	0.02	–	0.99
Date	**3.60**	**0.40**	**<0.01**	**5.10**	**0.07**	**<0.01**	**5.10**	**0.06**	**<0.01**
Block	–	–	–	0.43	–	0.67	0.29	–	0.77
Veg type x block	0.12	–	0.91	–	–	–	–	–	–
Veg type x date	1.25	–	0.21	**2.63**	**0.90**	**0.01**	**2.64**	**0.77**	**0.01**

^a^The *F* statistic is reported for the fixed effect of vegetation type; Wald’s Z is reported for all other random factors.

^b^Reported *P*-values are Bonferroni-corrected and boldface type highlights *P* < 0.05.

Temporal variation in microbial biomass was positively, but weakly associated with antecedent rainfall during the previous week (*r*^*2*^ = 0.044, *P* < 0.001, S3 Table).

There were no differences detected in microbial biomass among vegetation types (*P =* 0.657; Fig 1B) or block (Table 1).

### Bacteria taxonomic richness and phylogenetic diversity

Bacteria richness and phylogenetic diversity varied by date, with ~5-6x more taxa and ~3x higher PD per sample in the September 2012 date compared to other dates (*P* < 0.001, Fig 1C and 1E, Table 1 and S4 Table). Diversity patterns covaried with rainfall in the previous month (richness: *r*^*2*^ = 0.122, *P* < 0.001; PD: *r*^*2*^ = 0.130, *P* < 0.001), and soil NH_4_^+^ concentration (richness: *r*^*2*^ = 0.176, *P* < 0.001; PD: *r*^2^ = 0.168, *P* < 0.001; S3 Table). Changes in bacteria richness and PD among vegetation types also depended on sampling date (Table 1); *Hyeronima*, *Pentaclethra* and secondary forest stands had the highest richness and PD in the wetter September dates, while *Virola* and *Vochysia* also had the highest richness and PD in September 2012, but had lower richness and PD in September 2013 compared to February 2013.

**Fig 2:**
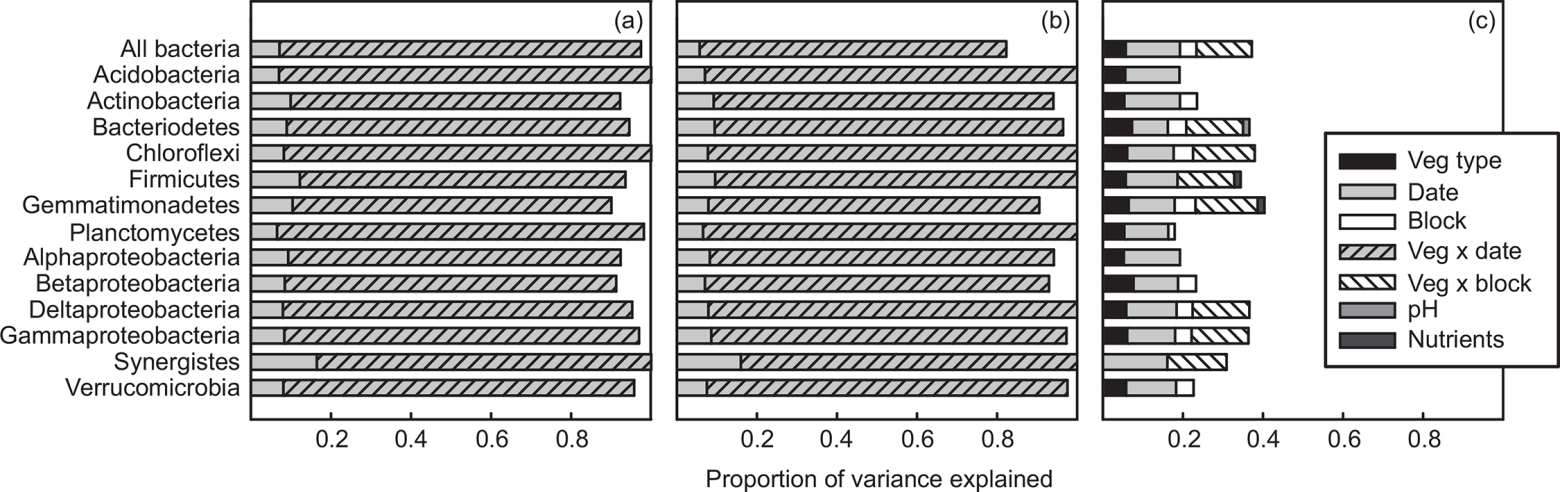
Variance decomposition of microbial diversity and composition. Proportion of variance explained in bacteria (a) richness, (b) phylogenetic diversity, and (c) composition by vegetation type (veg), date, block, and significant covariates for all bacteria and each bacteria phylum.

All bacteria phyla exhibited diversity patterns that were similar to the entire bacteria community, with richness and PD structured primarily by the interaction of vegetation type and date (80–93% variance explained; Fig 2A and 2B, S4 and S5 Tables).

### Bacteria community composition

**Fig 3:**
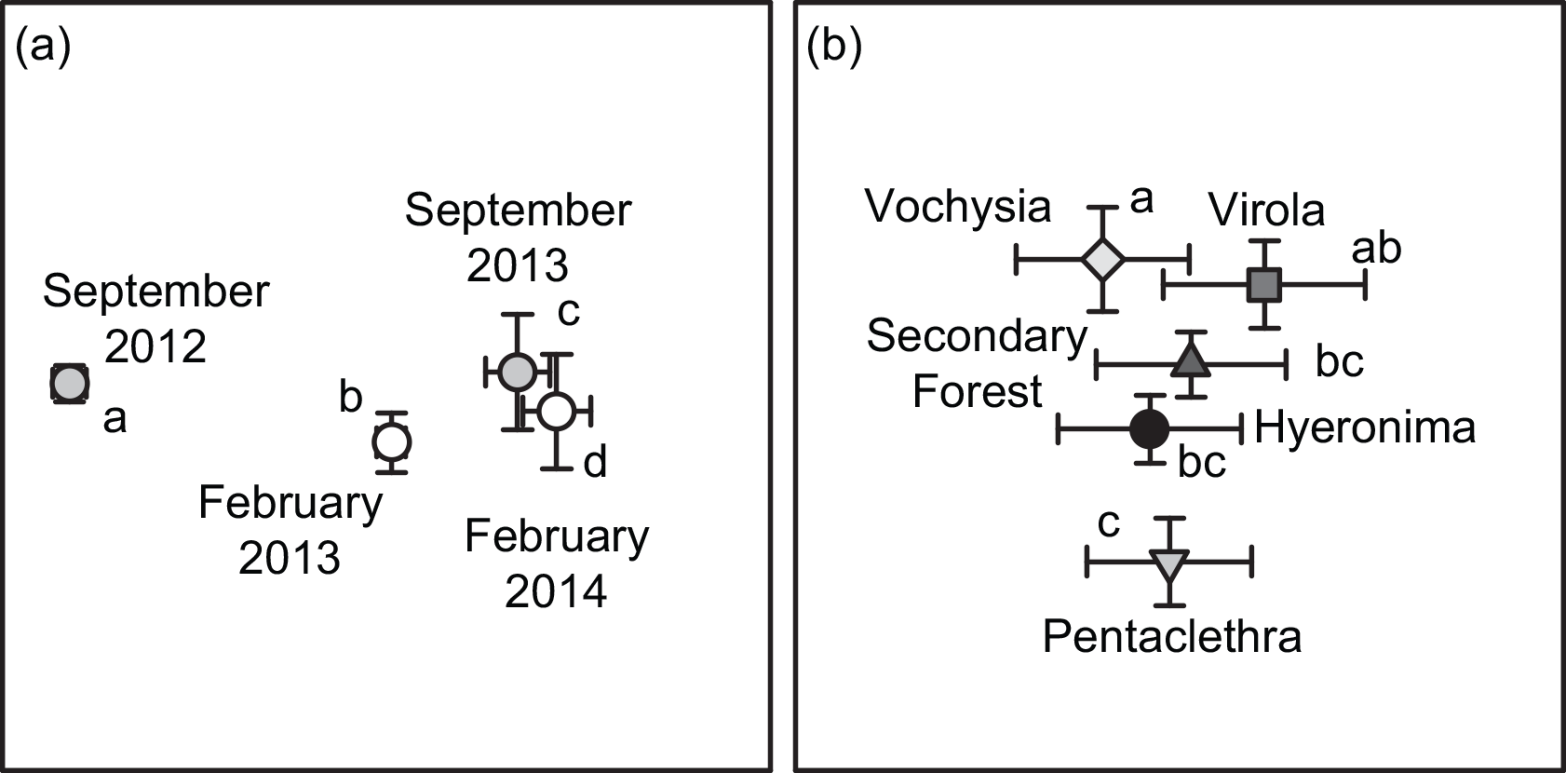
Effects of date and vegetation type on microbial community composition. NMS ordination of bacteria community composition by (a) date and (b) vegetation type. Data are presented as means ± 1 SE (n = 23 for date and 16 for vegetation type). Lowercase letters indicate significant differences among treatments based on PERMANOVA posthoc tests (*P* < 0.05 after Bonferroni correction).

Soil bacteria community composition was best explained by sampling date (*r*^2^ = 0.133; Table 2, Fig 2C), with significant differences among all dates (Fig 3A, S6 Table). The largest shifts occurred between the February 2014 and September 2012 sampling dates (Fig 3A). NMS Axis 1 was correlated with short-term rainfall in the previous week (*r*^*2*^ = 0.095, *P* < 0.001; S3 Table).

**Table 2:** PERMANOVA of bacterial community composition for all bacteria and separate phyla.

	All Bacteria			Acidobacteria			Actinobacteria			Bacteriodetes			Chloroflexi		
	*F*	*r*^*2*^	*P*	*F*	*r*^*2*^	*P*	*F*	*r*^*2*^	*P*	*F*	*r*^*2*^	*P*	*F*	*r*^*2*^	*P*
Veg type	**1.29**^a^	**0.06**	**<0.01**	**1.26**	**0.06**	**<0.01**	**1.23**	**0.06**	**0.02**	**1.60**	**0.08**	**<0.01**	**1.42**	**0.07**	**<0.01**
Date	**3.83**	**0.13**	**<0.01**	**3.81**	**0.13**	**<0.01**	**3.89**	**0.14**	**<0.01**	**2.44**	**0.09**	**<0.01**	**3.25**	**0.11**	**<0.01**
Block	**1.19**	**0.04**	**0.03**	1.14	0.04	0.09	**1.21**	**0.04**	**0.03**	**1.30**	**0.05**	**<0.01**	**1.43**	**0.05**	**<0.01**
Veg type x date	1.01	0.05	0.39	1.02	0.05	0.31	0.98	0.05	0.52	1.02	0.05	0.35	0.99	0.05	0.52
Veg type x block	**1.09**	**0.14**	**0.04**	1.07	0.14	0.07	1.04	0.13	0.20	**1.09**	**0.14**	**0.05**	**1.22**	**0.15**	**<0.01**
Soil pH^b^	–	–	NS	–	–	NS	–	–	NS	**1.30**	**0.02**	**0.04**	–	–	NS
PO_4_^-3^	–	–	NS	–	–	NS	–	–	NS	–	–	NS	–	–	NS
NO_3_^-^	–	–	NS	–	–	NS	–	–	NS	–	–	NS	–	–	NS
**Tot *r***^***2***^**, *P***	–	**0.37**	**<0.01**	–	**0.19**	**<0.01**	–	**0.24**	**<0.01**	–	**0.37**	**<0.01**	–	**0.38**	**<0.01**
	Firmicutes			Gemmatimonadetes			Planctomycetes			Alpha-Proteobacteria			Beta-Proteobacteria		
	*F*	*r*^*2*^	*P*	*F*	*r*^*2*^	*P*	*F*	*r*^*2*^	*P*	*F*	*r*^*2*^	*P*	*F*	*r*^*2*^	*P*
Veg type	**1.31**	**0.06**	**<0.01**	**1.50**	**0.07**	**<0.01**	**1.18**	**0.06**	**0.01**	**1.21**	**0.06**	**0.02**	**1.71**	**0.08**	**<0.01**
Date	**3.63**	**0.13**	**<0.01**	**3.30**	**0.11**	**<0.01**	**2.95**	**0.11**	**<0.01**	**3.89**	**0.14**	**<0.01**	**3.12**	**0.11**	**<0.01**
Block	1.16	0.04	0.05	**1.52**	**0.05**	**<0.01**	1.13	0.04	0.05	1.14	0.04	0.07	**1.25**	**0.04**	**<0.01**
Veg type x date	1.05	0.05	0.20	0.99	0.05	0.51	1.06	0.05	0.14	0.99	0.05	0.47	1.01	0.05	0.43
Veg type x block	**1.12**	**0.14**	**0.02**	**1.25**	**0.16**	**<0.01**	1.05	0.14	0.09	1.05	0.14	0.17	1.07	0.14	0.08
Soil pH	–	–	NS	–	–	NS	–	–	NS	–	–	NS	–	–	NS
PO_4_^-3^	–	–	NS	–	–	NS	**1.36**	**0.02**	**0.01**	–	–	NS	–	–	NS
NO_3_^-^	**1.31**	**0.02**	**0.05**	**1.49**	**0.02**	**0.02**	–	–	NS	–	–	NS	–	–	NS
**Tot *r***^***2***^**, *P***	–	**0.34**	**<0.01**	–	**0.40**	**<0.01**	–	**0.18**	**<0.01**	–	**0.19**	**<0.01**	–	**0.23**	**<0.01**
	Delta-Proteobacteria			Gamma-Proteobacteria			Synergistes			Verrucomicrobia					
	*F*	*r*^*2*^	*P*	*F*	*r*^*2*^	*P*	*F*	*r*^*2*^	*P*	*F*	*r*^*2*^	*P*			
Veg type	**1.32**	**0.06**	**<0.01**	**1.38**	**0.07**	**<0.01**	1.09	0.05	0.24	**1.25**	**0.06**	**<0.01**			
Date	**3.47**	**0.12**	**<0.01**	**3.28**	**0.12**	**<0.01**	**4.82**	**0.16**	**<0.01**	**3.51**	**0.12**	**<0.01**			
Block	**1.16**	**0.04**	**0.03**	**1.15**	**0.04**	**0.05**	1.03	0.04	0.38	**1.24**	**0.04**	**0.02**			
Veg type x date	1.00	0.05	0.46	0.99	0.05	0.54	0.99	0.04	0.48	0.95	0.05	0.76			
Veg type x block	**1.09**	**0.14**	**0.03**	**1.09**	**0.14**	**0.04**	**1.20**	**0.19**	**0.02**	1.07	0.14	0.10			
Soil pH	–	–	NS	–	–	NS	–	–	NS	–	–	NS			
PO_4_^-3^	–	–	NS	–	–	NS	–	–	NS	–	–	NS			
NO_3_^-^	–	–	NS	–	–	NS	–	–	NS	–	–	NS			
**Tot *r***^***2***^**, *P***	–	**0.37**	**<0.01**	–	**0.37**	**<0.01**	–	**0.31**	**<0.01**	–	**0.23**	**<0.01**			

^a^Bolded values are significant at *P* < 0.05 after Bonferroni correction.

^b^Only covariates that were significant in one or more of the models are displayed in the table.

Bacteria community composition also varied among vegetation types, but this variation explained little of the community variation over all (*r*^2^ = 0.060; Table 2). *Vochysia* and *Pentaclethra* stands varied the most in bacterial composition with all other vegetation types intermediate (Fig 3B). Soil pH partially mediated the effects of vegetation (S5 Table), with the lowest pH in *Pentaclethra* (4.20) soils, the highest pH in *Vochysia* soils (5.13), and intermediate pH in the remaining stands (4.69–4.76) that clustered together. Soil pH was also correlated with NMS Axis 2 (*r*^2^ = 0.130, *P* < 0.001; Fig 3B, S3 Table). The effects of vegetation varied with block, which explained more than twice the variation in bacteria community composition compared to vegetation type alone (*r*^2^ = 0.139; Table 2, Fig 2C). However, the majority of variation in bacteria community composition was unexplained by any of the factors we measured (*r*^2^ = 0.627). When bacteria phyla were analyzed separately, sampling date and vegetation-block interactions remained the main drivers of composition; however, phyla differed in how much ancillary variation in composition was explained by other factors (Fig 2C; Table 2).

### Soil enzyme activities

**Fig 4:**
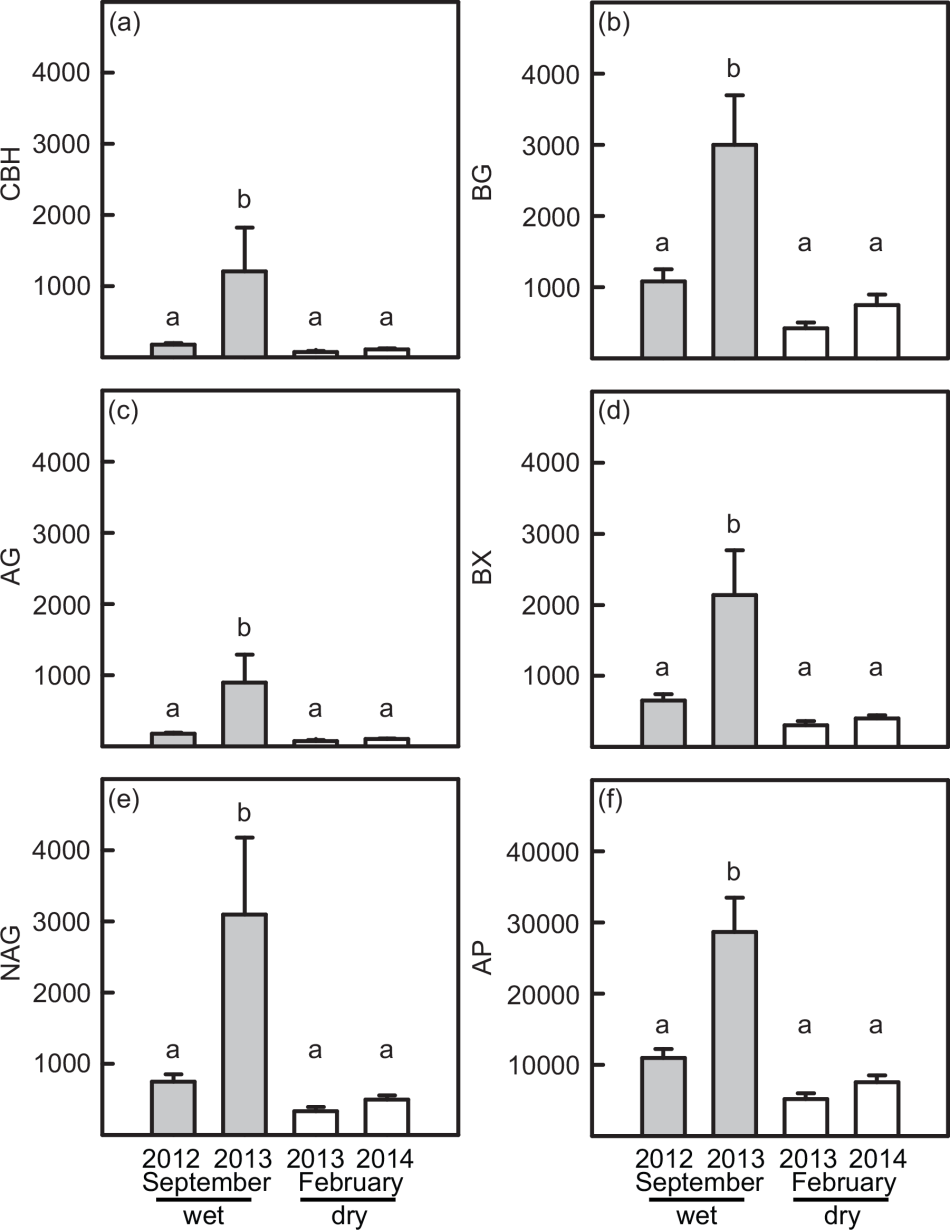
Enzyme activities (μmol^-1^ g dry soil^-1^ hour) for (a) CBH, (b) BG, (c) AG, (d) BX, (e) NAG, and (f) AP across vegetation types and sampling dates. Note the 10x scale difference for AP (f) compared to all other enzyme activities. Lowercase letters indicate significant differences among treatments in posthoc tests (*P* < 0.05 after Bonferroni correction). Error bars are ± 1 SE (n = 19).

All enzyme activities varied among dates (S7 Table). Enzyme activities in the wettest September 2013 samples were 5-12x greater than other dates on average, and activities varied by 200-850x across dates when all samples were considered (Fig 4). Acid phosphatase was the only enzyme that also exhibited a vegetation by date interaction (S7 Table). Acid phosphatase activities were the highest in all vegetation types in September 2013 and the lowest in February 2013, except *Hyeronima* stands, which contained the lowest enzyme activities in the February 2014 sampling date. Enzyme activities were also positively correlated with short-term antecedent rainfall (one week: *r*^*2*^ = 0.08–0.32, *P* < 0.05; one month: *r*^*2*^ = 0.06–0.29, *P* < 0.05) and with microbial biomass (*r*^*2*^ = 0.03–0.05) (S3 Table). Enzyme activities were not significantly affected by vegetation type or block (S7 Table).

## Discussion

We observed a large degree of temporal variation in soil bacteria biomass, function, richness, PD, composition, and structure independent of tree species that underscores the need to go beyond single time point studies in the tropics. Temporal variation is relatively common in ecosystems with stronger seasonality [58, 59], but the capacity of rainforest soil bacteria communities to respond to natural temporal climate and resource fluctuations was unknown. The observed temporal heterogeneity was partly related to changes in rainfall and soil resources over time, although these were not always consistent. Bacteria biomass and enzyme activities were highest in the wettest date (September 2013), whereas richness and PD were highest in the second-wettest date (September 2012) and correlated with soil NH_4_^+^. Bacteria community composition also tracked moisture in the previous week, with September 2012, being the most dissimilar from the other dates. The rainfall patterns observed in the current study are not atypical: precipitation at this site in February and September has differed by an average of 208 mm over the past 40 years (Organization for Tropical Studies Meteorological Database, 1963–2013). This result is nonetheless surprising given that soil moisture only ranged from ~63% to 75% across all dates. Thus, small differences in water availability from rain can have large belowground effects in this ecosystem, despite soils being consistently wet.

Based on our results, the two-fold shifts in precipitation expected with climate change [60] will exacerbate observed temporal heterogeneity in soil bacteria biomass, composition, and function. This is consistent with a prior rainfall exclusion experiment at the same site, where 15% reduction in field soil moisture resulted in subsequent increases in soil respiration compared to control soils at low moisture [61]. However, results from other studies are equivocal. For example, at two tropical forest sites less than 40 km apart, enzyme activities decreased by 50% in response to a 500% seasonal increase in precipitation and increased up to 140% in response to a 17% increase in soil moisture along a precipitation gradient [17, 62]. Such broad variation in responses may reflect complex processes in soil that are only partially mediated by moisture per se; for example, in [24] moisture was decoupled from the redox processes that drive microbial functions, such as decomposition. Progress in this area will require greater attention to the relationship between moisture, rainfall, and other factors such as soil texture that drive functional processes at microbial scales.

The experimental tree species also consistently affected soil bacteria community composition, including all phyla other than Synergistes. Discerning the effects of plant identity has historically been challenging in hyperdiverse rainforests, but this was made possible via experimental plots where soils were conditioned by monodominant tree species since 1988. In part, the observed tree species effects occurred because their soils differed in pH and nutrients. However, in contrast to our original hypothesis, composition also did not track previously observed differences in soil carbon inputs [28]. Many other studies have found that pH and nutrients are primary controllers of soil bacteria at local and global scales [63, 64], but other, under-reported, variables may also be important. For example, in a recent meta-analysis, tree species mainly affected concentrations of base cations and other micronutrients, but not microclimate, at the global scale [10]. All bacteria phyla also may not respond identically to plant-mediated belowground effects [65]. In the current study, only Bacteriodetes were significantly correlated with soil pH, whereas Firmicutes, Gemmatimonadetes and Planctomycetes were the only phyla associated with soil nutrients. One possibility is that variable plant soil feedbacks in tropical rainforests [66] may be partly mediated by both the large heterogeneity of plant inputs across species [18] and the sensitivity of underlying bacteria communities to these factors.

How tree species effects on soil bacteria will scale up to hyperdiverse tropical forests remains unknown, but the central position of the secondary forest plots in the NMS suggests an averaging effect of plant species on belowground bacteria community composition. This is further supported by the similarity in bacteria richness across vegetation types, which differs from the expectation of increased richness in secondary forest plots if tree species effects were additive. Many other studies have independently examined the effects of tree species identity [67] and plant diversity [68] on belowground soil bacteria, but none have simultaneously compared plant monocultures and mixtures over long time scales. In the second-longest experimental tree plantation and diversity manipulation of tropical tree species to date (~10 years) [3], distinct soil bacteria communities were found in monocultures compared to secondary forests without any averaging effects of plant diversity. This finding suggests that any averaging effects may be specific to the tree species being considered or develop over time scales longer than 10 years.

In addition to direct effects of tree species on bacteria, we observed both temporal and spatial heterogeneity in tree effects. Tree species effects on bacteria richness and PD varied by date and were correlated with NH_4_^+^, suggesting a role for temporal fluctuations in measured soil nutrients among the experimental stands. There also may be temporal variation in unmeasured factors related to tree phenology, such as root exudates and litter inputs [69]. Tree species effects on bacteria community composition were affected as much by spatial heterogeneity as by date. There was little spatial variation in soil nutrients, but heterogeneity among tree plots could be caused by variation in topography with slope and hilltop positions and associated differences in runoff, soil depth, and nutrients that are important for tree species inputs belowground [19, 70]. Non-dominant understory species that were not controlled in the experiment could also contribute to spatial patterns; however, 18 years after plots were established there was no significant variation in understory species across the vegetation types [30]. Spatially variable plant effects were also the main driver of soil fungal community composition in this system [27] and in regional dry forests [20], suggesting that spatial heterogeneity broadly affects tropical soil microbes.

The experimental tree stands at La Selva contained one of the most diverse communities of bacteria ever sampled at one geographic location, with more than 100,000 bacteria OTUs. This level of diversity may not be unique [71], particularly given the recent estimate of as many as 10^12^ bacterial taxa at the global scale [72]. However, links between high microbial species richness and function are still lacking for most ecosystems. Here we observed largely independent compositional and functional responses of bacteria to spatial and temporal heterogeneity. Bacteria community similarity based on Bray-Curtis was low, ranging from 5–28% among dates and 5–36% among experimental tree stands; yet enzyme activities that varied by 200-850x among samples were only weakly correlated with the NMS axis scores (*r*^2^ = 0.3–5.8%). These patterns likely reflect a combination of functional redundancy and plasticity in highly variable soil bacteria. The genes responsible for extracellular enzyme production are present throughout the entire bacteria phylogeny [73, 74], suggesting that many of the bacterial taxa in these soils were capable of producing the enzymes we measured. Therefore, physiological shifts in enzyme production [75] coupled with changes in diffusion of enzymes and substrates [76] among soils at different moistures can explain the majority of functional variation in this ecosystem. Ultimately, more refined functional assays of the entire microbial transcriptome or metabolome [77, 78], will be necessary to fully understand how microbial species diversity and function are linked across spatial and temporal scales in belowground tropical ecosystems.

### Conclusion

We demonstrated that the perceived dependence of tropical soil bacteria on tropical plant identity is only partial, with large contributions from temporal and spatial heterogeneity. Given that bacteria responded to the interactions of tree species with both date and block, changes that exacerbate heterogeneity, such as expected future increases in seasonality and drought [60, 79, 80], may have direct consequences for these belowground ecosystems. Further experimental work will be needed to determine the optimal temporal and spatial scales at which to study tropical soil bacteria.

## Supporting Information

S1 Table Soil moisture and climate data for all vegetation types in all dates. (PDF)

S2 Table Soil characteristics from all vegetation types in all dates (PDF)

S3 Table Bivariate correlations (Pearson *r*) for unmanipulated factors in the experiment. (PDF)

S4 Table Linear mixed model of bacterial richness by phylum. (PDF)

S5 Table Linear mixed model of bacteria PD by phylum. (PDF)

S6 Table Pairwise posthoc comparisons among vegetation types and dates for the PERMANOVA. (PDF)

S7 Table Linear mixed model of enzyme activities. (PDF)
