# Supplementary material for: Temporal and Spatial Variation of Soil Bacteria Richness, Composition, and Function in a Neotropical Rainforest
Source: PLoS One. 2016 Jul 8;11(7):e0159131. doi: 10.1371/journal.pone.0159131 (PMC4938164; doi:10.1371/journal.pone.0159131)
Supplement: S1 Table — (PDF) [file pone.0159131.s001.pdf]

S1 Table. Previous 1 week, 1 month, 3 months and 6 months rainfall for each sampling date. Values are presented with means and posthoc designations by date when significant at $P < 0.05$. Soil moisture (Wald's Z = 4.466, P < 0.001), previous 1 week rainfall ($F_{1,3} = 34.745$, $P < 0.001$), and 1 month rainfall ($F_{1,3} = 523.962$, $P < 0.001$) were higher in the September sampling dates, while previous 3 months' rainfall ($F_{1,3} = 8.492$, $P = 0.004$), and 6 months' rainfall ($F_{1,3} = 5.109$, $P = 0.026$) were highest in 2013.

Sampling Date	Soil moisture	1 Week Rainfall	1 Month Rainfall	3 Month Rainfall	6 Month Rainfall
Sep 2012	73.06^{a} (1.91)	34.03^{b}	256.28^{a}	912.86^{ab}	1636.71^{ab}
Feb 2013	67.17^{b} (1.70)	19.81^{b}	73.66^{b}	1239.26^{a}	1951.73^{a}
Sep 2013	73.86^{a} (1.74)	63.76^{a}	304.03^{a}	1207.84^{a}	1969.60^{a}
Feb 2014	67.73^{b} (1.28)	44.20^{a}	157.48^{b}	559.57^{b}	1452.37^{b}
