# Supplementary material for: Temporal and Spatial Variation of Soil Bacteria Richness, Composition, and Function in a Neotropical Rainforest
Source: PLoS One. 2016 Jul 8;11(7):e0159131. doi: 10.1371/journal.pone.0159131 (PMC4938164; doi:10.1371/journal.pone.0159131)
Supplement: S2 Table — (PDF) [file pone.0159131.s002.pdf]

S2 Table. Soil characteristics from all vegetation types in all dates. Values are presented with means (1 SE) and posthoc letter designations by date or vegetation type (for pH only) when $P < 0.05$ in linear mixed model analyses. Soil PO_4^{-3} concentrations differed over time (Wald's Z = 4.476, $P < 0.001$), as did NH_4^+ concentrations (Wald's Z = 4.526, $P < 0.001$), with both being highest in the wetter, September sampling dates. Soil NO_3^- concentrations also differed over time (Wald's Z = 4.502, $P < 0.001$), with the drier, February sampling dates having the highest concentrations. Soil moisture was highest in the wetter sampling point in September, 2012 and lowest in the drier sampling point in February, 2014 (Wald's Z =4.446, $P < 0.001$). Soil PO_4^{-3} (Wald's Z = 2.410, $P = 0.016$), NH_4^+ (Wald's Z = 2.395, $P = 0.017$) and NO_3^- also had significant vegetation type x date interactions.

	**pH**				**Soil moisture**				**PO_4^{-3} (mg kg^{-1})**			
**Date**	**September^{a}**		**February^{b}**		**September^{a}**		**February^{b}**		**September^{a}**		**February^{b}**	
**Vegetation Type**	**2012**	**2013**	**2013**	**2014**	**2012**	**2013**	**2013**	**2014**	**2012**	**2013**	**2013**	**2014**
Hyeronima	4.37^{b}	4.43^{b}	4.38^{b}	3.94^{b}	77.14	78.01	74.38	69.45	2.34	4.38	2.26	2.25
	(0.05)	(0.07)	(0.05)	(0.11)	(4.60)	(1.45)	(3.60)	(3.05)	(0.47)	(0.76)	(0.28)	(0.12)
Pentaclethra	4.09^{c}	4.25^{c}	4.17^{c}	4.22^{c}	62.01	63.16	61.26	61.25	2.34	4.33	2.24	2.15
	(0.02)	(0.09)	(0.07)	(0.08)	(2.00)	(2.69)	(3.81)	(3.00)	(0.33)	(0.63)	(0.36)	(0.15)
Virola	4.55^{abc}	4.48^{abc}	4.47^{abc}	4.00^{abc}	70.95	75.44	71.36	65.51	2.38	4.98	2.65	2.28
	(0.17)	(0.06)	(0.06)	(0.10)	(3.29)	(3.67)	(2.20)	(3.93)	(0.45)	(0.88)	(0.28)	(0.13)
Vochysia	4.83^{a}	4.78^{a}	4.74^{a}	4.16^{a}	77.76	74.96	61.19	64.07	1.95	3.87	1.85	2.11
	(0.02)	(0.06)	(0.11)	(0.13)	(4.45)	(4.09)	(0.01)	(2.23)	(0.47)	(0.41)	(0.24)	(0.08)
Secondary Forest	4.44^{ab}	4.49^{ab}	4.44^{ab}	4.42^{ab}	76.03	72.57	66.72	65.59	2.46	4.42	2.47	2.30
	(0.03)	(0.05)	(0.06)	(0.09)	(1.78)	(1.67)	(1.25)	(3.38)	(0.45)	(0.50)	(0.26)	(0.18)

	**NH_4^+ (mg kg^{-1})**				**NO_3^- (mg kg^{-1})**			
**Date**	**September^{a}**		**February^{b}**		**September^{b}**		**February^{a}**	
**Vegetation Type**	**2012**	**2013**	**2013**	**2014**	**2012**	**2013**	**2013**	**2014**
Hyeronima	12.83	12.77	5.74	5.69	0.30	0.10	2.48	2.54
	(2.21)	(0.69)	(1.82)	(0.33)	(0.25)	(0.05)	(0.61)	(1.26)
Pentaclethra	12.07	10.01	3.71	4.24	1.55	1.48	8.37	2.28
	(2.20)	(1.38)	(0.81)	(0.31)	(0.87)	(0.60)	(3.23)	(0.84)
Virola	11.56	11.86	8.21	5.88	0.31	0.08	1.58	1.58
	(1.64)	(0.29)	(1.84)	(0.99)	(0.26)	(0.03)	(0.89)	(0.60)
Vochysia	13.01	14.35	4.89	6.08	0.05	0.05	2.07	0.32
	(2.07)	(0.85)	(2.66)	(0.29)	(4.91^{-18})	(4.90^{-18})	(1.34)	(0.17)
Secondary Forest	11.51	12.21	4.33	5.34	0.29	0.53	4.56	3.82
	(2.44)	(0.65)	(1.22)	(0.48)	(0.24)	(0.48)	(0.70)	(1.16)
