# Supplementary material for: Temporal and Spatial Variation of Soil Bacteria Richness, Composition, and Function in a Neotropical Rainforest
Source: PLoS One. 2016 Jul 8;11(7):e0159131. doi: 10.1371/journal.pone.0159131 (PMC4938164; doi:10.1371/journal.pone.0159131)
Supplement: S4 Table — (PDF) [file pone.0159131.s004.pdf]

S4 Table. Linear mixed model of bacterial richness for all phyla as a function of vegetation type, sampling date, block and interactions between vegetation type and block and vegetation type and date. Bold indicates *P* < 0.05 after Bonferroni correction.

			**Vegetation Type**			**Date**			**Vegetation Type * Date**			**Block**			**Vegetation Type * Block**		
	Num df	Denom df	*F*	*P*	R^2	Wald's Z	*P*	R^2	Wald's Z	*P*	R^2	Wald's Z	*P*	R^2	Wald's Z	*P*	R^2
Acidobacteria	4	15.039	0.039	0.997	–	**5.246**	**< 0.001**	**0.071**	**2.633**	**0.008**	**0.929**	–	–	–	–	–	–
Actinobacteria	4	15.060	0.058	0.993	–	**5.927**	**< 0.001**	**0.100**	**2.574**	**0.010**	**0.823**	0.874	0.382	–	–	–	–
Bacteriodetes	4	15.129	0.375	0.823	–	**6.271**	**< 0.001**	**0.090**	**2.598**	**0.009**	**0.856**	0.704	0.481	–	–	–	–
Chloroflexi	4	15.069	0.083	0.986	–	**5.247**	**< 0.001**	**0.083**	**2.615**	**0.009**	**0.917**	–	–	–	–	–	–
Firmicutes	4	15.112	0.094	0.983	–	**5.220**	**< 0.001**	**0.123**	**2.576**	**0.10**	**0.813**	–	–	–	0.009	0.993	–
Gemmatimonadetes	4	16.769	0.085	0.986	–	**5.113**	**< 0.001**	**0.105**	**2.594**	**0.009**	**0.796**	–	–	–	1.259	0.208	–
Planctomycetes	4	15.022	0.068	0.991	–	**5.105**	**< 0.001**	**0.066**	**2.640**	**0.008**	**0.916**	0.177	0.859	–	–	–	–
Alpha-Proteobacteria	4	15.130	0.034	0.998	–	**6.460**	**< 0.001**	**0.094**	**2.591**	**0.010**	**0.831**	0.855	0.393	–	–	–	–
Beta-Proteobacteria	4	15.099	0.124	0.972	–	**4.542**	**< 0.001**	**0.086**	**2.611**	**0.009**	**0.828**	0.501	0.617	–	0.187	0.852	–
Delta-Proteobacteria	4	15.197	0.049	0.995	–	**4.544**	**< 0.001**	**0.081**	**2.625**	**0.009**	**0.873**	–	–	–	0.360	0.719	–
Gamma-Proteobacteria	4	14.996	0.038	0.997	–	**6.477**	**<0.001**	**0.084**	**2.609**	**0.009**	**0.886**	0.729	0.466	–	–	–	–
Synergistes	4	15.118	0.055	0.994	–	**5.250**	**< 0.001**	**0.166**	**2.463**	**0.014**	**0.834**	–	–	–	–	–	–
Verrucomicrobia	4	15.016	0.088	0.985	–	**5.102**	**< 0.001**	**0.082**	**2.609**	**0.009**	**0.876**	0.683	0.495	–	–	–	–
