# Supplementary material for: Temporal and Spatial Variation of Soil Bacteria Richness, Composition, and Function in a Neotropical Rainforest
Source: PLoS One. 2016 Jul 8;11(7):e0159131. doi: 10.1371/journal.pone.0159131 (PMC4938164; doi:10.1371/journal.pone.0159131)
Supplement: S5 Table — (PDF) [file pone.0159131.s005.pdf]

S5 Table. Linear mixed model of bacteria PD for all phyla as a function of vegetation type, sampling date, block and interactions between vegetation type and block and vegetation type and date. Bold indicates *P* < 0.05 after Bonferroni correction.

	**Vegetation Type**					**Date**			**Vegetation Type * Date**			**Block**			**Vegetation Type * Block**		
	Num df	Denom df	*F*	*P*	R^2	Wald's Z	*P*	R^2	Wald's Z	*P*	R^2	Wald's Z	*P*	R^2	Wald's Z	*P*	R^2
Acidobacteria	4	15.035	0.030	0.998	–	**5.246**	**< 0.001**	**0.071**	**2.633**	**0.008**	**0.929**	–	–	–	–	–	–
Actinobacteria	4	15.052	0.049	0.995	–	**6.467**	**< 0.001**	**0.093**	**2.590**	**0.010**	**0.848**	0.803	0.422	–	–	–	–
Bacteriodetes	4	15.129	0.201	0.934	–	**6.156**	**< 0.001**	**0.095**	**2.598**	**0.009**	**0.870**	0.370	0.711	–	–	–	–
Chloroflexi	4	15.050	0.056	0.994	–	**5.246**	**< 0.001**	**0.078**	**2.622**	**0.009**	**0.922**	–	–	–	–	–	–
Firmicutes	4	15.405	0.059	0.993	–	**6.502**	**< 0.001**	**0.096**	**2.627**	**0.009**	**0.904**	–	–	–	–	–	–
Gemmatimonadetes	4	17.132	0.050	0.995	–	**6.052**	**< 0.001**	**0.079**	**2.640**	**0.008**	**0.827**	–	–	–	1.552	0.121	–
Plantomycetes	4	15.019	0.047	0.995	–	**5.245**	**< 0.001**	**0.066**	**2.640**	**0.008**	**0.934**	–	–	–	–	–	–
Alpha-Proteobacteria	4	15.043	0.02	0.999	–	**6.587**	**< 0.001**	**0.083**	**2.608**	**0.009**	**0.859**	0.898	0.369	–	–	–	–
Beta-Proteobacteria	4	15.198	0.119	0.974	–	**4.565**	**< 0.001**	**0.071**	**2.637**	**0.008**	**0.859**	0.321	0.748	–	0.340	0.734	–
Delta-Proteobacteria	4	15.030	0.026	0.999	–	**5.246**	**< 0.001**	**0.079**	**2.620**	**0.009**	**0.921**	–	–	–	–	–	–
Gamma-Proteobacteria	4	15.029	0.026	0.999	–	**5.102**	**< 0.001**	**0.087**	**2.605**	**0.009**	**0.887**	0.304	0.761	–	–	–	–
Synergistes	4	15.083	0.045	0.996	–	**5.252**	**< 0.001**	**0.160**	**2.473**	**0.013**	**0.840**	–	–	–	–	–	–
Verrucomicrobia	4	15.015	0.076	0.989	–	**5.103**	**< 0.001**	**0.076**	**2.622**	**0.009**	**0.900**	0.347	0.728	–	–	–	–
