# Supplementary material for: Temporal and Spatial Variation of Soil Bacteria Richness, Composition, and Function in a Neotropical Rainforest
Source: PLoS One. 2016 Jul 8;11(7):e0159131. doi: 10.1371/journal.pone.0159131 (PMC4938164; doi:10.1371/journal.pone.0159131)
Supplement: S6 Table — (PDF) [file pone.0159131.s006.pdf]

S6 Table. Pairwise posthoc comparisons among vegetation types and dates for the PERMANOVA analysis of bacteria community composition. Bolded values are significant at $P < 0.05$ after Bonferroni correction.

	**All Bacteria**	
	r^2	*P*
**Vegetation Type**		
Hyeronima v. *Pentaclethra*	0.035	0.0.079
Hyeronima v. *Virola*	0.032	0.132
Hyeronima v. *Vochysia*	**0.048**	**0.014**
Hyeronima v. Secondary Forest	0.030	0.205
Pentaclethra v. *Virola*	**0.039**	**0.021**
Pentaclethra v. *Vochysia*	**0.056**	**0.003**
Pentaclethra v. Secondary Forest	0.035	0.057
Virola v. *Vochysia*	**0.041**	**0.047**
Virola v. Secondary Forest	0.030	0.218
Vochysia v. Secondary Forest	0.041	0.073
**Date**		
September 2012 v. February 2013	**0.153**	**< 0.001**
September 2012 v. September 2013	**0.138**	**< 0.001**
September 2012 v. February 2014	**0.146**	**< 0.001**
February 2013 v. September 2013	**0.051**	**< 0.001**
February 2013 v. February 2014	**0.045**	**< 0.001**
September 2013 v. February 2014	**0.031**	**< 0.001**
