# Supplementary material for: Temporal and Spatial Variation of Soil Bacteria Richness, Composition, and Function in a Neotropical Rainforest
Source: PLoS One. 2016 Jul 8;11(7):e0159131. doi: 10.1371/journal.pone.0159131 (PMC4938164; doi:10.1371/journal.pone.0159131)
Supplement: S7 Table — (PDF) [file pone.0159131.s007.pdf]

S7 Table. Linear mixed model of enzyme activities as a function of vegetation type, sampling date, block and the interactions of vegetation type and date and vegetation type and block. Bolded values are significant after Bonferroni correction at *P* < 0.05. F statistic reported for fixed effects of vegetation type; Wald's Z reported for all other random factors. For the fixed effect of vegetation type, df = 4; df are not calculated for random covariance parameters in the LMM.

	**AG**			**BG**			**BX**		
	F or Wald's Z	r^2	*P*	*F or Wald's Z*	r^2	*P*	*F or Wald's Z*	r^2	*P*
Vegetation type	0.597	—	0.670	0.519	—	0.723	0.385	—	0.815
Date	**4.231**	**0.852**	**< 0.001**	**3.588**	**0.600**	**<0.001**	**4.579**	**0.772**	**< 0.001**
Block	0.409	—	0.682	—	—	—	0.088	—	0.930
Vegetation type x block	—	—	—	0.689	—	0.491	0.366	—	0.714
Vegetation type x date	0.803	—	0.422	1.600	—	0.097	1.263	—	0.207

	**CBH**			**NAG**			**AP**		
	F or Wald's Z	r^2	*P*	*F or Wald's Z*	r^2	*P*	*F or Wald's Z*	r^2	*P*
Vegetation type	0.629	—	0.649	0.528	—	0.718	0.339	—	0.848
Date	**4.281**	**0.886**	**< 0.001**	**3.744**	**0.781**	**< 0.001**	**4.548**	**0.494**	**< 0.001**
Block	0.500	—	0.617	0.240	—	0.810	0.725	—	0.469
Vegetation type x block	—	—	—	0.016	—	0.987	0.728	—	0.467
Vegetation type x date	0.545	—	0.586	1.176	—	0.240	**2.028**	**0.386**	**< 0.001**
