# Supplementary material for: Temporal and Spatial Variation of Soil Bacteria Richness, Composition, and Function in a Neotropical Rainforest
Source: PLoS One. 2016 Jul 8;11(7):e0159131. doi: 10.1371/journal.pone.0159131 (PMC4938164; doi:10.1371/journal.pone.0159131)

S3 Table. Bivariate correlations (Pearson r) for unmanipulated factors in the experiment. Abbreviations for enzymes are as follows: CBH:cellobiohydrolase, BG:beta-glucosidase, AG:alpha-glucosidase, NAG:N-acetyl-glucosaminidase, BX:beta-xylosidase, and AP:acid phosphatase. Bolded values were significant at *P* < 0.05 after Bonferroni correction.

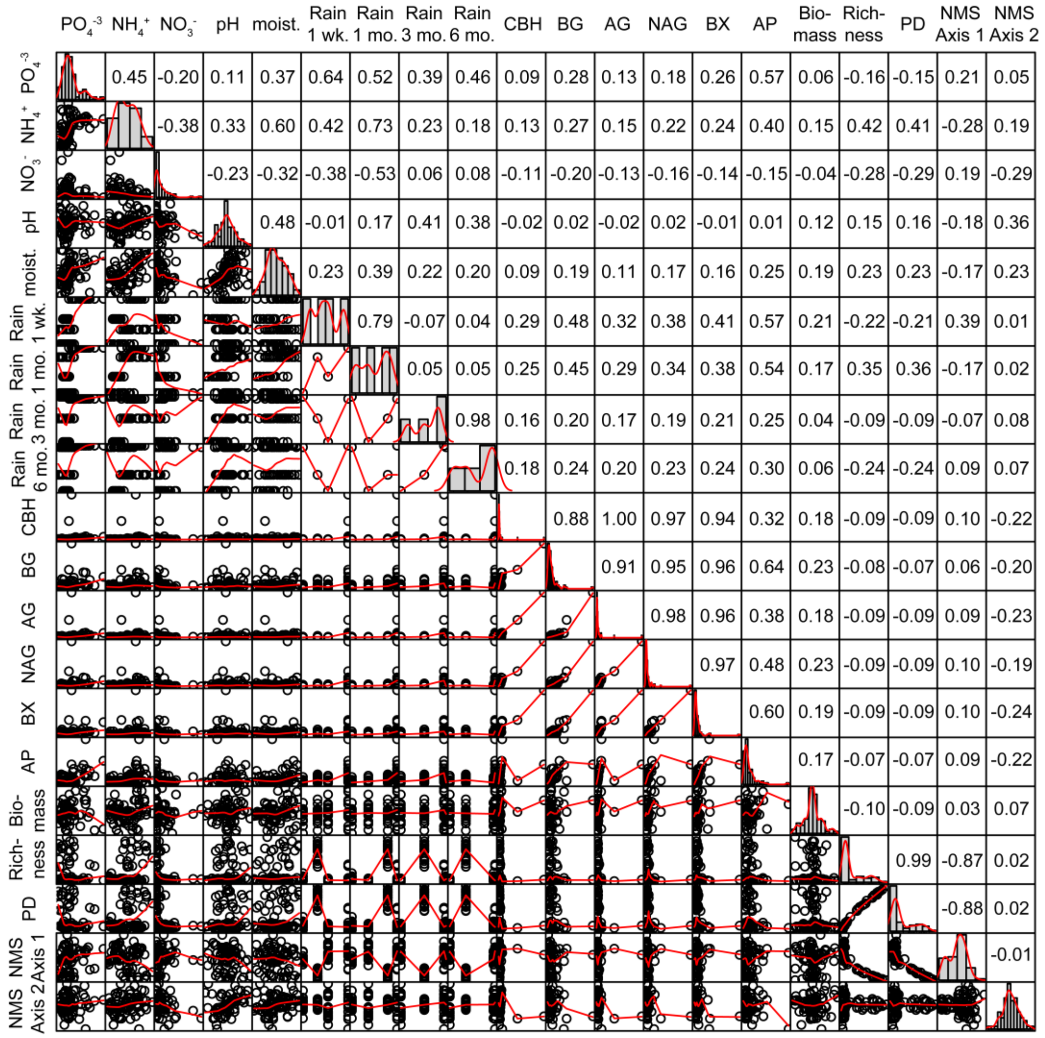

Supplement: S3 Table — (PDF) [file pone.0159131.s003.pdf]
